# Bis(η^3^-2-*tert*-butyl-1-trimethyl­silyl-3-phenyl-1-aza­all­yl)nickel(II)

**DOI:** 10.1107/S1600536811009469

**Published:** 2011-03-19

**Authors:** Junsheng Hao, Haimei Liu, Hai-Yan Yuan

**Affiliations:** a School of Chemistry and Chemical Engineering, Shanxi University, Taiyuan 030006, People’s Republic of China; b Institute of Applied Chemistry, Shanxi University, Taiyuan 030006, People’s Republic of China

## Abstract

The title compound, [Ni(C_15_H_24_NSi)_2_], is a homoleptic metal–η^3^-aza­allyl centrosymmetric complex containing two aza­allyl ligands bound in an η^3^-manner to an Ni^II^ atom located on a center of symmetry. The overall coordination about the Ni^II^ atom is square-planar. The C and N atoms of the aza­allyl group are *sp* ^2^-hybridized. The uneven Ni—C and Ni—N distances [2.045 (5)/2.060 (6) and 1.916 (5) Å] are influenced by a steric hindering effect from the nearby benzene ring.

## Related literature

For metal-mediated reactions, see: Blystone (1989 ▶). For related 1-aza­allyl complexes including some main group elements and transition metals, see: Avent *et al.* (2004 ▶); Caro *et al.* (2001 ▶); Hitchcock *et al.* (2000 ▶). For related cobalt–η^3^-allyl complexes, see: Yuan *et al.* (2007 ▶).
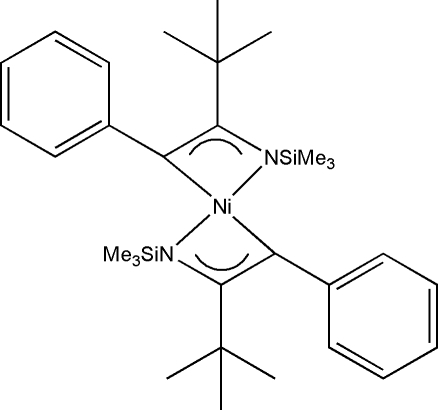


## Experimental

### 

#### Crystal data

[Ni(C_15_H_24_NSi)_2_]
*M* *_r_* = 551.59
Monoclinic, 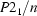

*a* = 10.309 (6) Å
*b* = 9.289 (6) Å
*c* = 16.521 (9) Å
β = 94.84 (2)°
*V* = 1576.4 (16) Å^3^
*Z* = 2
Mo *K*α radiation
μ = 0.71 mm^−1^
*T* = 213 K
0.30 × 0.30 × 0.20 mm

#### Data collection

Siemens SMART CCD area-detector diffractometer
Absorption correction: multi-scan (*SADABS*; Bruker, 2009 ▶) *T*_min_ = 0.815, *T*_max_ = 0.871
7488 measured reflections
2776 independent reflections
2446 reflections with *I* > 2σ(*I*)
*R*_int_ = 0.061

#### Refinement

*R*[*F* ^2^ > 2σ(*F* ^2^)] = 0.096
*wR*(*F* ^2^) = 0.186
*S* = 1.36
2776 reflections
166 parameters
H-atom parameters constrained
Δρ_max_ = 0.63 e Å^−3^
Δρ_min_ = −1.22 e Å^−3^

### 

Data collection: *SMART* (Bruker, 2009 ▶); cell refinement: *SAINT* (Bruker, 2009 ▶); data reduction: *SAINT*; program(s) used to solve structure: *SHELXS97* (Sheldrick, 2008 ▶); program(s) used to refine structure: *SHELXL97* (Sheldrick, 2008 ▶); molecular graphics: *SHELXTL* (Sheldrick, 2008 ▶); software used to prepare material for publication: *SHELXL97*.

## Supplementary Material

Crystal structure: contains datablocks global, I. DOI: 10.1107/S1600536811009469/jj2080sup1.cif

Structure factors: contains datablocks I. DOI: 10.1107/S1600536811009469/jj2080Isup2.hkl

Additional supplementary materials:  crystallographic information; 3D view; checkCIF report

## Figures and Tables

**Fig. 1.**
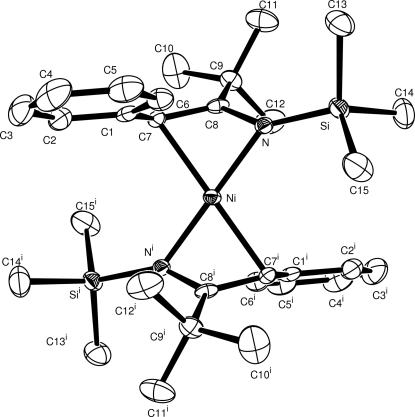
View of the title molecule showing the atom-labelling scheme. Displacement ellipsoids are drawn at the 30% probability level. H atoms are omitted for clarity. The symmetry operator for i is –X, –Y, –Z.

## supplementary crystallographic information

### Comment

Metal-η^3^-allyl complexes are well known to play an important role in many metal-mediated reactions (Blystone *et al.*, 1989). Lappert and co-workers have prepared a variety of 1-azaallyl complexes containing main group elements and transition metals (Avent *et al.*, 2004; Caro *et al.*, 2001; Hitchcock *et al.*, 2000). Recently, Yuan and co-workers have prepared related Cobalt η^3^-allyl complexes (Yuan *et al.*, 2007). As part of an subsequent investigation of metal-η^3^-azaallyl complexes, we have prepared the title complex, [Ni(C_30_H_48_N_2_Si_2_)_2_], (I),

The title compound is a homoleptic metal-η^3^-azaallyl centrosymmetric complex containing two azaallyl ligands bound in an η^3^ manner to a Ni^II^ atom located at the center of symmetry, thereby, forming two nonplanar 4-membered rings, N/C8/C7/Ni (Fig. 1). The dihedral angle between the N/C7/Ni and C8/C7/Ni planes is 49.0 (3)°.  The C and N atoms of the azaallyl group are *sp*^2^– hybridized with the N—C8 bond [1.355 (7) Å] showing double-bond character. The uneven Ni—C7, Ni—C8 and Ni—N distances [2.045 (5), 2.060 (6)Å, and 1.916 (5)Å] are influenced by a steric hindered effect from the nearby benzene ring (C1—C6).

### Experimental

All manipulations were carried out under argon or *in vacuo* using standard Schlenk techniques. The title complex was synthesized according to literature methods (Hitchcock *et al.*, 2000; Avent *et al.*, 2004). To a solution of trimethylsilylmethyltolulithium (6 mmol) in diethyl ether(20 ml), *tert*-butyl nitrile (6 mmol) was added at *ca* 273 K and the solution was stirred for 15 min and then for 5 h at room temperature. To this solution, NiCl_2_(3 mmol) was added at *ca* 200 K and the suspension was stirred for 15 min and then for 5 h at room temperature. The mixture was filtered and the filtrate was carefully concentrated under a vacuum until yellow crystals of the title compound appeared.

### Refinement

All H atoms were positioned geometrically, with CH = 0.96– 0.98 Å, CH_3_ = 0.96Å and refined as riding, allowing for free rotation of the methyl groups. The *U*_iso_(H) values were set at 1.18-1.21 *U*_eq_(CH) or 1.5*U*_eq_(CH_3_).

### Crystal data

**Table:**

[Ni(C_15_H_24_NSi)_2_]	*F*(000) = 596
*M**_r_* = 551.59	*D*_x_ = 1.162 Mg m^−^^3^
Monoclinic, *P*2_1_/*n*	Mo *K*α radiation, λ = 0.71073 Å
Hall symbol: -P 2yn	Cell parameters from 1405 reflections
*a* = 10.309 (6) Å	θ = 2.4–26.7°
*b* = 9.289 (6) Å	µ = 0.71 mm^−^^1^
*c* = 16.521 (9) Å	*T* = 213 K
β = 94.84 (2)°	Block, yellow
*V* = 1576.4 (16) Å^3^	0.30 × 0.30 × 0.20 mm
*Z* = 2	

### Data collection

**Table:**

Siemens SMART CCD area-detector diffractometer	2776 independent reflections
Radiation source: fine-focus sealed tube	2446 reflections with *I* > 2σ(*I*)
graphite	*R*_int_ = 0.061
φ and ω scans	θ_max_ = 25.0°, θ_min_ = 2.3°
Absorption correction: multi-scan (*SADABS*; Bruker, 2009)	*h* = −12→9
*T*_min_ = 0.815, *T*_max_ = 0.871	*k* = −9→11
7488 measured reflections	*l* = −19→19

### Refinement

**Table:**

Refinement on *F*^2^	Primary atom site location: structure-invariant direct methods
Least-squares matrix: full	Secondary atom site location: difference Fourier map
*R*[*F*^2^ > 2σ(*F*^2^)] = 0.096	Hydrogen site location: inferred from neighbouring sites
*wR*(*F*^2^) = 0.186	H-atom parameters constrained
*S* = 1.36	*w* = 1/[σ^2^(*F*_o_^2^) + (0.0426*P*)^2^ + 3.0952*P*] where *P* = (*F*_o_^2^ + 2*F*_c_^2^)/3
2776 reflections	(Δ/σ)_max_ < 0.001
166 parameters	Δρ_max_ = 0.63 e Å^−^^3^
0 restraints	Δρ_min_ = −1.22 e Å^−^^3^

### Special details

**Table:**

Geometry. All e.s.d.'s (except the e.s.d. in the dihedral angle between two l.s. planes) are estimated using the full covariance matrix. The cell e.s.d.'s are taken into account individually in the estimation of e.s.d.'s in distances, angles and torsion angles; correlations between e.s.d.'s in cell parameters are only used when they are defined by crystal symmetry. An approximate (isotropic) treatment of cell e.s.d.'s is used for estimating e.s.d.'s involving l.s. planes.
Refinement. Refinement of *F*^2^ against ALL reflections. The weighted *R*-factor *wR* and goodness of fit *S* are based on *F*^2^, conventional *R*-factors *R* are based on *F*, with *F* set to zero for negative *F*^2^. The threshold expression of *F*^2^ > σ(*F*^2^) is used only for calculating *R*-factors(gt) *etc*. and is not relevant to the choice of reflections for refinement. *R*-factors based on *F*^2^ are statistically about twice as large as those based on *F*, and *R*- factors based on ALL data will be even larger.

### Fractional atomic coordinates and isotropic or equivalent isotropic displacement parameters (Å^2^)

**Table:**

	*x*	*y*	*z*	*U*_iso_*/*U*_eq_	
Ni	0.5000	0.5000	0.0000	0.0230 (3)	
N	0.5562 (5)	0.5466 (5)	0.1104 (3)	0.0258 (11)	
Si	0.60753 (18)	0.6959 (2)	0.16563 (10)	0.0343 (5)	
C1	0.6907 (6)	0.2919 (7)	0.0403 (4)	0.0319 (15)	
C2	0.7162 (8)	0.1682 (7)	−0.0031 (4)	0.0461 (18)	
H2	0.6477	0.1078	−0.0208	0.055*	
C3	0.8420 (9)	0.1330 (10)	−0.0205 (5)	0.067 (3)	
H3	0.8575	0.0502	−0.0498	0.080*	
C4	0.9421 (9)	0.2215 (11)	0.0060 (6)	0.071 (3)	
H4	1.0265	0.1984	−0.0052	0.086*	
C5	0.9199 (7)	0.3447 (9)	0.0491 (5)	0.057 (2)	
H5	0.9887	0.4047	0.0670	0.068*	
C6	0.7934 (7)	0.3781 (7)	0.0655 (4)	0.0404 (17)	
H6	0.7783	0.4615	0.0944	0.048*	
C7	0.5535 (6)	0.3120 (6)	0.0600 (3)	0.0288 (14)	
H7	0.4961	0.2299	0.0465	0.035*	
C8	0.5045 (6)	0.4128 (6)	0.1139 (3)	0.0264 (13)	
C9	0.3895 (6)	0.3787 (7)	0.1648 (4)	0.0354 (15)	
C10	0.3392 (9)	0.2272 (9)	0.1507 (6)	0.079 (3)	
H10A	0.2748	0.2066	0.1878	0.119*	
H10B	0.4100	0.1603	0.1594	0.119*	
H10C	0.3009	0.2184	0.0959	0.119*	
C11	0.4382 (8)	0.3936 (10)	0.2540 (4)	0.064 (2)	
H11A	0.3679	0.3754	0.2872	0.096*	
H11B	0.4706	0.4894	0.2641	0.096*	
H11C	0.5069	0.3255	0.2671	0.096*	
C12	0.2786 (7)	0.4838 (9)	0.1442 (5)	0.063 (2)	
H12A	0.2472	0.4728	0.0881	0.095*	
H12B	0.3093	0.5805	0.1534	0.095*	
H12C	0.2092	0.4646	0.1779	0.095*	
C13	0.7233 (8)	0.6372 (9)	0.2517 (4)	0.059 (2)	
H13A	0.6765	0.5887	0.2914	0.088*	
H13B	0.7669	0.7198	0.2760	0.088*	
H13C	0.7864	0.5728	0.2320	0.088*	
C14	0.4802 (8)	0.8102 (8)	0.2082 (5)	0.063 (2)	
H14A	0.4187	0.8418	0.1650	0.094*	
H14B	0.5206	0.8925	0.2350	0.094*	
H14C	0.4358	0.7551	0.2465	0.094*	
C15	0.6989 (9)	0.8097 (9)	0.0976 (5)	0.066 (3)	
H15A	0.7616	0.7517	0.0727	0.099*	
H15B	0.7430	0.8852	0.1286	0.099*	
H15C	0.6394	0.8512	0.0562	0.099*	

### Atomic displacement parameters (Å^2^)

**Table:**

	*U*^11^	*U*^22^	*U*^33^	*U*^12^	*U*^13^	*U*^23^
Ni	0.0255 (6)	0.0245 (6)	0.0189 (5)	−0.0018 (5)	0.0021 (4)	0.0027 (5)
N	0.029 (3)	0.027 (3)	0.022 (2)	0.007 (2)	0.006 (2)	0.003 (2)
Si	0.0386 (11)	0.0359 (10)	0.0281 (9)	−0.0032 (8)	0.0005 (8)	−0.0048 (8)
C1	0.035 (4)	0.033 (4)	0.028 (3)	0.007 (3)	0.003 (3)	0.014 (3)
C2	0.057 (5)	0.040 (4)	0.042 (4)	0.011 (4)	0.008 (4)	0.007 (3)
C3	0.074 (7)	0.058 (6)	0.072 (6)	0.030 (5)	0.025 (5)	0.008 (5)
C4	0.048 (6)	0.082 (7)	0.088 (7)	0.032 (5)	0.028 (5)	0.027 (6)
C5	0.035 (5)	0.074 (6)	0.061 (5)	0.007 (4)	0.003 (4)	0.019 (4)
C6	0.038 (4)	0.046 (4)	0.038 (4)	0.001 (3)	0.006 (3)	0.008 (3)
C7	0.041 (4)	0.020 (3)	0.025 (3)	−0.003 (3)	−0.001 (3)	0.004 (3)
C8	0.022 (3)	0.030 (3)	0.027 (3)	−0.008 (3)	−0.002 (3)	0.013 (3)
C9	0.033 (4)	0.043 (4)	0.031 (3)	−0.007 (3)	0.010 (3)	0.002 (3)
C10	0.090 (7)	0.067 (6)	0.088 (7)	−0.036 (5)	0.054 (6)	−0.007 (5)
C11	0.058 (5)	0.099 (7)	0.037 (4)	0.002 (5)	0.016 (4)	0.013 (4)
C12	0.043 (5)	0.079 (6)	0.070 (5)	0.003 (4)	0.022 (4)	0.028 (5)
C13	0.061 (6)	0.067 (5)	0.046 (4)	−0.006 (4)	−0.012 (4)	−0.005 (4)
C14	0.067 (6)	0.054 (5)	0.066 (5)	0.012 (4)	−0.001 (4)	−0.022 (4)
C15	0.089 (7)	0.062 (5)	0.048 (5)	−0.043 (5)	0.007 (4)	−0.001 (4)

### Geometric parameters (Å, °)

**Table:**

Ni—N	1.916 (5)	C7—H7	0.9800
Ni—N^i^	1.916 (5)	C8—C9	1.544 (8)
Ni—C8	2.045 (5)	C9—C10	1.511 (10)
Ni—C8^i^	2.045 (5)	C9—C12	1.521 (10)
Ni—C7^i^	2.060 (6)	C9—C11	1.523 (9)
Ni—C7	2.060 (6)	C10—H10A	0.9600
N—C8	1.355 (7)	C10—H10B	0.9600
N—Si	1.720 (5)	C10—H10C	0.9600
Si—C15	1.857 (7)	C11—H11A	0.9600
Si—C13	1.860 (8)	C11—H11B	0.9600
Si—C14	1.871 (7)	C11—H11C	0.9600
C1—C6	1.365 (9)	C12—H12A	0.9600
C1—C2	1.390 (9)	C12—H12B	0.9600
C1—C7	1.490 (8)	C12—H12C	0.9600
C2—C3	1.390 (10)	C13—H13A	0.9600
C2—H2	0.9300	C13—H13B	0.9600
C3—C4	1.362 (12)	C13—H13C	0.9600
C3—H3	0.9300	C14—H14A	0.9600
C4—C5	1.377 (11)	C14—H14B	0.9600
C4—H4	0.9300	C14—H14C	0.9600
C5—C6	1.389 (9)	C15—H15A	0.9600
C5—H5	0.9300	C15—H15B	0.9600
C6—H6	0.9300	C15—H15C	0.9600
C7—C8	1.414 (8)		
			
N—Ni—N^i^	180.0	N—C8—C7	114.7 (5)
N—Ni—C8	39.8 (2)	N—C8—C9	122.3 (5)
N^i^—Ni—C8	140.2 (2)	C7—C8—C9	122.6 (5)
N—Ni—C8^i^	140.2 (2)	N—C8—Ni	64.9 (3)
N^i^—Ni—C8^i^	39.8 (2)	C7—C8—Ni	70.4 (3)
C8—Ni—C8^i^	180.0	C9—C8—Ni	128.6 (4)
N—Ni—C7^i^	108.3 (2)	C10—C9—C12	108.8 (7)
N^i^—Ni—C7^i^	71.7 (2)	C10—C9—C11	108.4 (6)
C8—Ni—C7^i^	139.7 (2)	C12—C9—C11	109.8 (6)
C8^i^—Ni—C7^i^	40.3 (2)	C10—C9—C8	112.1 (5)
N—Ni—C7	71.7 (2)	C12—C9—C8	110.1 (5)
N^i^—Ni—C7	108.3 (2)	C11—C9—C8	107.7 (5)
C8—Ni—C7	40.3 (2)	C9—C10—H10A	109.5
C8^i^—Ni—C7	139.7 (2)	C9—C10—H10B	109.5
C7^i^—Ni—C7	180.0	H10A—C10—H10B	109.5
C8—N—Si	145.2 (4)	C9—C10—H10C	109.5
C8—N—Ni	75.2 (3)	H10A—C10—H10C	109.5
Si—N—Ni	137.9 (3)	H10B—C10—H10C	109.5
N—Si—C15	106.7 (3)	C9—C11—H11A	109.5
N—Si—C13	108.5 (3)	C9—C11—H11B	109.5
C15—Si—C13	107.7 (4)	H11A—C11—H11B	109.5
N—Si—C14	117.6 (3)	C9—C11—H11C	109.5
C15—Si—C14	108.2 (4)	H11A—C11—H11C	109.5
C13—Si—C14	107.7 (4)	H11B—C11—H11C	109.5
C6—C1—C2	117.8 (6)	C9—C12—H12A	109.5
C6—C1—C7	125.8 (6)	C9—C12—H12B	109.5
C2—C1—C7	116.2 (6)	H12A—C12—H12B	109.5
C1—C2—C3	121.4 (8)	C9—C12—H12C	109.5
C1—C2—H2	119.3	H12A—C12—H12C	109.5
C3—C2—H2	119.3	H12B—C12—H12C	109.5
C4—C3—C2	119.1 (8)	Si—C13—H13A	109.5
C4—C3—H3	120.5	Si—C13—H13B	109.5
C2—C3—H3	120.5	H13A—C13—H13B	109.5
C3—C4—C5	120.9 (8)	Si—C13—H13C	109.5
C3—C4—H4	119.6	H13A—C13—H13C	109.5
C5—C4—H4	119.6	H13B—C13—H13C	109.5
C4—C5—C6	119.1 (8)	Si—C14—H14A	109.5
C4—C5—H5	120.5	Si—C14—H14B	109.5
C6—C5—H5	120.5	H14A—C14—H14B	109.5
C1—C6—C5	121.8 (7)	Si—C14—H14C	109.5
C1—C6—H6	119.1	H14A—C14—H14C	109.5
C5—C6—H6	119.1	H14B—C14—H14C	109.5
C8—C7—C1	128.1 (5)	Si—C15—H15A	109.5
C8—C7—Ni	69.3 (3)	Si—C15—H15B	109.5
C1—C7—Ni	102.8 (4)	H15A—C15—H15B	109.5
C8—C7—H7	114.7	Si—C15—H15C	109.5
C1—C7—H7	114.7	H15A—C15—H15C	109.5
Ni—C7—H7	114.7	H15B—C15—H15C	109.5

Symmetry codes: (i) −*x*+1, −*y*+1, −*z*.
